# Acetylcholinesterase enzyme among cancer patients a potential diagnostic and prognostic indicator a multicenter case–control study

**DOI:** 10.1038/s41598-024-55604-6

**Published:** 2024-03-01

**Authors:** Gasmelseed Y. Ahmed, Ahmed A. Osman, Ahlam Mukhtar

**Affiliations:** 1 Columbia University Hospital, New York, NY USA; 2 Faculty of Medicine, and Health Sciences, Managil University for Sciences & Technology, Managil, Sudan; 3 Independant Scholar, Chesterfield, UK; 4 Stack Laboratory, Federal Ministry of Health, Khartoum, Sudan; 5 Radiation and Isotopes Center Khartoum, Federal Ministry of Health, Khartoum, Sudan

**Keywords:** Acetylcholinesterase enzyme (AChE), Acetylcholine (ACh), Cancer, Sudan, Cancer, Environmental sciences

## Abstract

Acetylcholinesterase enzyme (AChE) activity is impaired by a variety of inhibitors including organophosphorus pesticides, leading to the accumulation of acetylcholine. In this study, we aimed to determine the association between cancer and the blood level of the (AChE). This is a multicenter hospital-based case–control study conducted in the Radiation and Isotopes Center Khartoum, and Institute of Nuclear Medicine and Molecular Biology and Oncology Gezira. One hundred and fifty participants, half of them cancer patients and half cancer free were recruited. All participants were screened for demographic, environmental, occupational, and clinical characteristics. Blood for the (AChE) activity test was drawn from participants in the two groups. The mean age of the participants was 40.6 ± 14.8 years. Geographical distribution showed the Central Region of Sudan had the highest rate of cancer, followed by North State, Khartoum State, West State, and East State. The most common tumor subtype was breast cancer, followed by leukemia, colon, esophageal, and prostate cancer. Inferential analysis revealed significantly impaired (AChE) activity among cancer patients compared to controls (53.4 ± 20.3% vs. 93.8 ± 8.8, *p*-value 0.001). There was a significant statistical association between impaired (AChE) activity and cancer. (AChE) activity might be applied in the future as a diagnostic biomarker and therapeutic target. Further large sample and molecular studies are recommended.

## Introduction

The etiology of cancer involves several causes where cells start to grow abnormally in an uncontrollable manner. Cancer cells tend to invade adjoining parts of the body and diffuse to affect other organs. It is the second cause of death with nearly 10 million deaths worldwide, the lung cancer is the leading cause of death followed by colorectal, liver, stomach, and breast cancers^1^. Only 5–10% of all cancer cases can be attributed to genetic defects, whereas the remaining 90–95% have their roots in the environment and lifestyle, including cigarette smoking, fried foods, red meat, alcohol, sun exposure, environmental pollutants, infections, stress, obesity, and physical inactivity^2^. In most cases, genetic mutations are present while the associated risk factors start the process of changing the normal pattern of cellular proliferation and differentiation. Scholars identified genes such as oncogenes, tumor suppressor genes, apoptosis genes, and DNA repair genes to be responsible for mutations and changing the normal cell division processes^3,4^. (AChE) plays an important role in the formation of apoptosome and the regulation of the apoptotic process^5^. Decrease levels of (AChE) produce substantial DNA damage, reactive oxygen stress (ROS), and rise micronuclei frequencies, leading to the increased risk of genotoxic processes^6,7^. Carcinogenesis analysis showed the fundamental role of endogenous oxidative damage to DNA, which is prevented by continuous cell repair processes. The rate of cell division is the important indicator of the carcinogenesis process, and it is obviously influenced by cytotoxicity, hormonal release, growth, and inflammatory processes involving cells and organs. Moreover, the rate of cell division ascertains the possibility of altering DNA lesions to mutations^8^. (AChE) is involved in the termination of impulse transmission by hydrolysis of acetylcholine. The enzyme inactivation induced by various inhibitors, some toxicological and others pharmacological, leads to acetylcholine accumulation. The inhibition of (AChE) is presently a common pharmacological approach for some diseases, including; Alzheimer’s disease (AD), some eye diseases, postoperative use, and myasthenia gravis. While nontherapeutic inhibition is mainly caused by exposure to organophosphorus (OP) compound^9,10^. Acetylcholine is one of the important chemicals present in the central nervous system (CNS). The name "acetylcholine" comes from its structural formula composed of acetic acid ester and choline. Naturally, choline is found in foods such as liver, egg yolks, vegetables seeds, and legumes. When choline enters the blood circulation, it can cross the blood–brain barrier and uptake by cholinergic nerve terminals to formulate acetylcholine. Choline acetyltransferase (CAT) induces the reaction of choline with acetyl-CoA to yield acetylcholine^11,12^. (AChE) is considered as a cholinergic enzyme present in postsynaptic neuromuscular junctions that prompt the divide of acetylcholine (ACh) into acetic acid and choline^13,14^. Tumor markers are a group of substances that indicate the carcinogenic status of the body, and they include cytoplasmic proteins, hormones, cell surface antigens, receptors, enzymes, oncofetal antigens, oncogenes, and their products. They had several clinical implementations, as they’re applied in cancer diagnosis, prediction of therapeutic response, cases management, monitoring, and prognosis^15^. Currently, in clinical practice settings, tumor markers play an important role in cancer management. The biomarker identification is mainly held by histopathology tests for tissues, body fluids, or blood^16^. Applying biomarkers in oncology plays an important preventive function in screening and early diagnosis of malignancy in apparently asymptomatic people to properly manage patients and prevent further possible complications^17^. Furthermore, biomarkers have a wide range of benefits as they are used in medicine, environmental health, toxicology, developmental biology, and basic scientific research. Biological monitoring plays a crucial role in occupational health and risk assessments. In recent decades, biomarker use has gained attention in occupational and environmental medicine. This interest accompanied the development of human biomonitoring. Scholars introduced the concept of human biomonitoring to monitor the chemicals or biomarkers in the fluids or tissues of persons who had occupational exposure to chemical, physical, or biological risk factors or in the general environment. (AChE) is a specific validated biomarker measuring the effect of pesticides and has had a major role in environmental and occupational medicine^18,19^. AChE may function as a potential biomarker for cancer diagnosis or prognosis^20^. A recent epidemiological cancer study of World Health Organization and American Cancer Society database reports the current incremental trend of cancer prevalence and mortality expected in the next 40 years, suggesting that the burden will remain as the first and foremost public health issue which poses a dramatic clinical burden, disruption of social standards, and erosion of economic resources^21^. A study conducted in South Africa evaluated 65 medicinal plants used for CNS and memory-related disorders; 30 of them have been defined as (AChE) inhibitors, with the most active compounds isolated from Crinum moorei*,* Scadoxus puniceus, and Acacia nilotica^22^*.* In Sudan, organophosphorus (OP) compounds applied in agriculture is a major (AChE) inhibitor, where the laws and policies governing pesticide use are outdated and deficient for protection of workers and consumers^23^. Sudan is experiencing a growing cancer problem, but little is known about the tumor patterns, cancer epidemiology, and ethnic or environmental risk factors behind this increase^24^. There is public concern among the Sudanese community about the heavy use of agricultural chemicals in the country, assuming an association with the steady increase in the rate of cancer, with the question of whether it is related to the impaired (AChE) resulting from the agricultural and industrial occupational hazards. In this study, we aimed to explore this public health problem and evaluate the association between impaired AChE and cancer*.*

## Methods

This is a multicenter hospital-based case–control study conducted at the Radiation and Isotopes Center Khartoum and the Institute of Nuclear Medicine and Molecular Biology and Oncology Gezira, Sudan. A validated questionnaire was applied to collect sociodemographic and clinical variables from all participants, beside data on (AChE) of cases, and baseline information on medical history of controls.

Inclusion and exclusion criteria: Participants included as cases with confirmed diagnosis of cancer, inpatients receiving treatment in the cancer centers, and controls who were free of cancer. Participants excluded: if severely ill, cases or controls with Alzheimer disease, and cases or controls taking (AChE) inhibitor medication.

**Figure 1:**
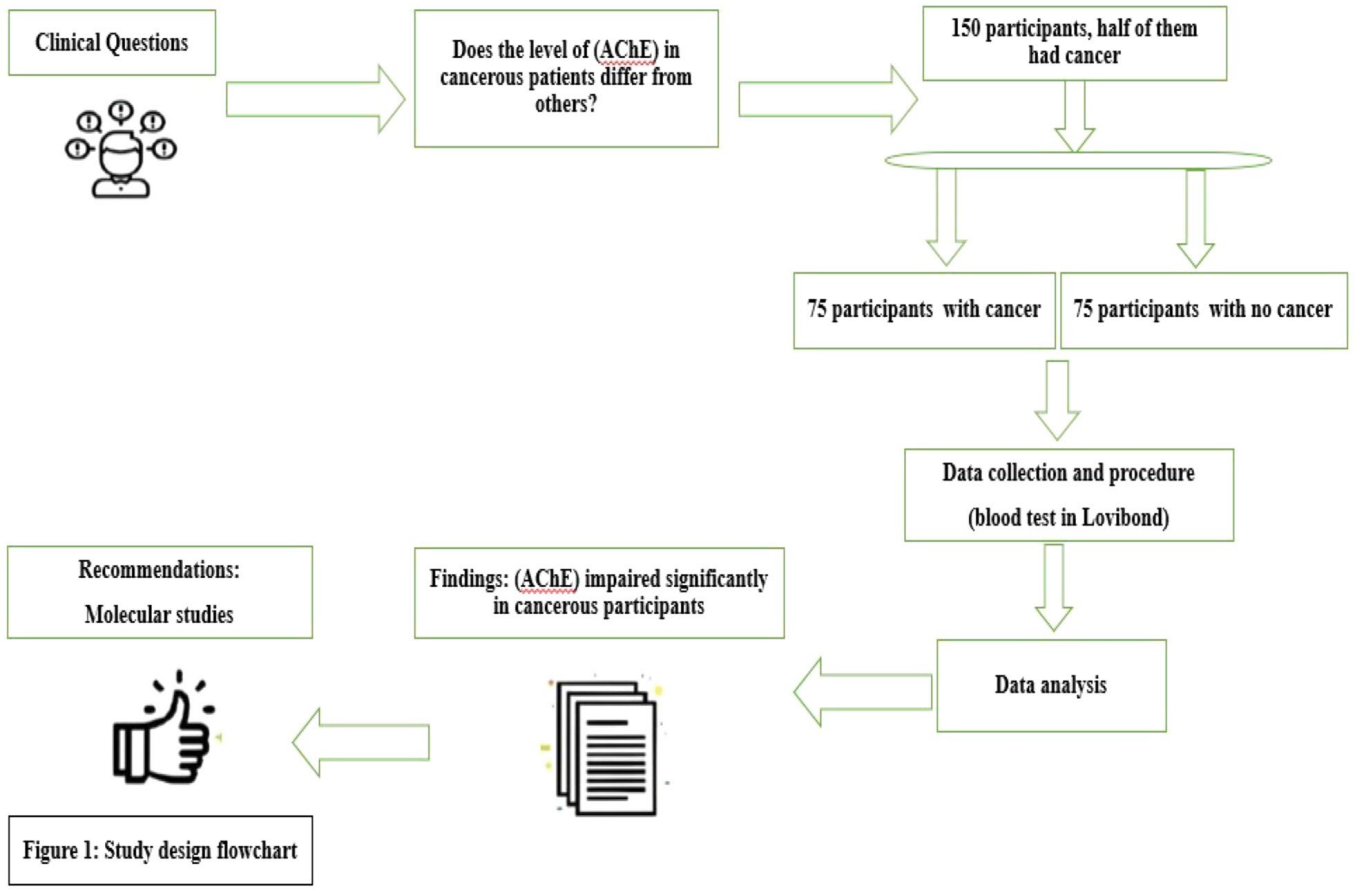
Flow chart for the study design; impaired (AChE) in cancer makes it a key diagnostic indicator: A hospital-based multicenter case–control study; 2020 (n = 150).

Sample size calculation and sampling techniques: A relevant reference supporting and justifying the sample size calculation for this study was the Malaysian study “Norsyazwani M. et al.” which reported a mean and standard deviation of blood cholinesterase in exposed people of (86.7 ± 13.5)^25^. Applying their effect size with assumptions of 95% level of confidence and 3.1 margin of error in the standard formula [n = (z^2^*s^2^/e^2^)], the optimal sample of 75 participants per group was estimated. We identified charts for cancer patients at the two cities of the study, assigned serial numbers for each, then selected a random sample of 75 patients. All the cases were confirmed for cancer as documented in the hospitals’ records with diagnosis based on the standard clinical, histopathological, imaging diagnostic tools, and tumor biomarkers screening. A control group of seventy-five non-cancer participants were chosen from family members, relatives, friends, and visitors of the patients. Their selection was matched as 1:1 paring for region of residence, age, and occupation (Fig. 1).

Preparation of reagents and testing blood (AChE) activity: Blood samples to test acetylcholinesterase level were drawn from all patients and controls at time of the test. A finger stick was applied on every participant to obtain only one drop of blood sample, followed by addition of reagent which develops a color in the blood sample ranges from green to yellow depending on the level of acetylcholinesterase activity. We applied the Lovibond machine for measurements of the participants’ blood (AChE) percentage of activities (Fig. 1). The Lovibond calorimetric test provided rapid results with sufficient accuracy. One drop of blood was collected from each participant. Following the addition of the reagent, a color in the sample develops depending on the level of (AChE) activity. The sample matches with glass color standards in a test disc using a comparator^26,27^. A laboratory toxicology specialist prepared the reagent; (acetylcholine perchlorate and bromothymol blue) and conducted the blood test through the following three steps: First, water was prepared by distillation followed by boil and cooling, stored in a tightly closed bottle to prevent absorption of atmospheric carbon dioxide. Ethanol was prepared, and a substrate solution containing 0.25 mg of acetylcholine perchlorate dissolved in 50 ml water was prepared freshly each day that the test is performed. Bromothymol blue indicator solution containing 112 mg of the sodium salt of Bromothymol blue per 250 ml of water, was prepared 24 h before use to ensure it is completely dissolved. Finally, the solution became dark green with a final pH range of 5.8–6.6. One (1.0 ml) of water pipette was placed into a clean test tube and added to 10 µl (0.01ml) of a blood sample. The solution was transferred to a 2.5 mm cell and the cell was placed in the left-hand compartment of the comparator. This solution acted as a blood blank and was used for all samples for one day. Then, 0.5 ml of indicator solution was added to a clean test tube, and 10 µl of the blood of the control person was added carefully and shaken gently. Then, 0.5 ml of substrate solution was added and immediately the mixture was transferred to a clean 2.5 mm cell and placed in the right-hand compartment of the comparator. The reading of the results was carried out using daylight to find the glass standard that most closely matched the sample color and record the corresponding cholinesterase activity (when reading 0 − 12.5 on the scale, the reagent solutions were considered correct) and a clean test tube was reserved for each blood sample. To each tube 0.5 ml of Bromothymol blue indicator solution was added, each blood sample was homogenized by gentle shaking, and 10 µl of blood was added carefully to the respective test tube. Starting the stopwatch when pipetting the substrate solution into the first tube, 0.5 ml of substrate solution was added to each test tube at exactly two-minute intervals, and after (20–30 min) the time that the control sample reached 100%. Reading was repeated for the others for the same time then the results were read; enzymatic activity of 75–100% was normal, 50–70% mildly impaired, and 25–50% was severely impaired, while 0–25% was a serious condition^25,28,29^.

### Statistical analysis

We applied a standard statistical procedure through the Statistical Package for Social Sciences SPSS (IBM 2020. Corporation, Armonk, NY, USA). The collected data validated for missing values and accuracy before conducting the statistical analysis. We have performed descriptive and inferential statistics: descriptive analysis was conducted for categorical variables such as sociodemographic characteristics and (AChE) activity (impaired versus normal) and reported as frequencies, and for continuous variables reported means ± standard deviation (SD). In inferential statistics, we have applied univariate and multivariate statistics and reported Fisher’s exact test, independent t-tests, and OR with *p*-values. Results were considered statistically significant for a two-sided *p*-value ≤ 0.05.

### Ethics approval and consent to participate

The study was conducted in accordance with the guidelines of the Declaration of Helsinki and approved by the Institutional Review Board of the Sudanese Ministry of Health and College of Medicine University of Khartoum, Sudan. Informed consent was obtained from all study participation and/or parent/legal guardian involved in the study.

## Results

**Figure 2:**
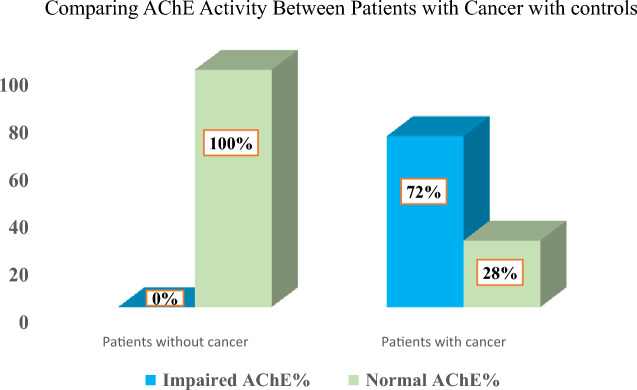
Measurements of (AChE) activity among the two groups; 2020 (n = 150). (AChE) activity impaired significantly in patients with cancer *p*-value = 0.0001.

Of the total one hundred and fifty patients, 75 (50%) were diagnosed with cancer 21 (28%) of them on chemotherapy. The other half were cancer-free with a mean age of 40.6 ± 14.8 years. Geographical distribution showed the central region of Sudan had the highest rate of cancer, followed by the northern state, Khartoum, and western and eastern states. We found more than one-third of the participants had only elementary schooling. The most common tumor subtype was breast cancer, followed by leukemia, colon, esophageal, and prostate cancers. 28 (18.7%) of the participants live within chemical exposure zone majority of them were cases (Table 1). The results showed variant levels of enzyme impairment in all cancer subtypes; the least impairment was noticed in endometrial, cervical, and breast cancers, and the most severely impaired were the liver, pancreas, and colon cancer, (Table 2). Univariate analysis compared individual cancers versus controls, then compared the mean AChE versus controls, independent t-tests yielded (AChE) activity significantly lower in cancerous patients compared to controls (*p*-value range between 0.02 to 0.0001), (Tables 2 and 3). A cut-off 75% for normal (AChE) revealed cases having significantly lower (AChE) activity compared to controls, *p*-value for Fisher’s exact test 0.0001, (Fig. 2). Multivariate analysis applying binary regression showed males having 30% higher risk of cancer, while the matched age has no difference OR 1.3 and 1.006 respectively, (Table 4).

**Table 1:** Basic demographics and clinical characteristics, Impaired (AChE) in cancer makes it a key diagnostic indicator: A hospital-based multicenter case–control study; 2020 (n = 150).

Characteristics	N (%)
Gender	
Male	79 (52.7%)
Female	71(47.3%)
Educational status	
Illiterate	18 (12.0%)
Elementary school	68 (45.4%)
High school	38 (25.3%)
College	26 (17.3%)
Residence	
Central Sudan	59 (39.3%)
North Sudan	49 (32.7%)
Khartoum	21 (14.0%)
West Sudan	12 (8.0%)
East Sudan	09 (6.0%)
Occupation	
Farmers	50 (33.3%)
Housewife	40 (26.7%)
Workers	37 (24.7%)
Govrnmental jobs	23 (15.3%)
Live in or close to farm	54 (36.0%)
Live close to industries	64 (42.7%)
Live close to both	28 (18.7%)
Medical history	
Cancer	75 (50%)
DM	15 (10.0%)
Hypertension	12 (8.0%)
Chronic kidney diseases (CKD)	7 (4.6%)
Others	13 (8.7%)
No diseases	28 (18.7%)
Type of cancer	
Breast	13 (17.3%)
Leukemia	11 (14.7%)
Colon	10 (13.3%)
Esophegus	9 (12.0%)
Prostate	8 (10.7%)
Cervix	6 (8.0%)
Lymphoma	5 (6.7%)
Ovary	4 (5.3%)
Liver	3 (4.0%)
Nasopharyngeal	2 (2.7%)
Endometrial	2 (2.7%)
Pancreas	2 (2.7%)
On chemotherapy	
Yes	54 (72.0%)
No	21 (28..%)
AchE activity	
Normal	96 (64.0%)
Impaired (all among cancer patients)	54 (36.0%)
Age	40.6 ± 14.8 years

**Table 2:** Level of impaired enzyme per cancer subtype, Impaired (AChE) in cancer makes it a key diagnostic indicator: A hospital-based multicenter case control study; 2020 (n = 150).

Cancer subtype	Mean AChE in cancer	*p*-value
Endometrial	75.0 ± 0.01	0.0001
Cervix	72.1 ± 7.9	0.0181
Breast	68.3 ± 14.9	0.0001
Prostate	62.5 ± 16.4	0.0043
Ovary	59.4 ± 23.7	0.0001
Leukemia	57.3 ± 12.8	0.0001
Lymphoma	57.6 ± 6.8	0.0246
Nasopharyngeal	56.2 ± 8.8	0.0001
Esophageal	40.3 ± 5.5	0.0001
Colon	30.0 ± 14.7	0.0001
Pancreas	25.0 ± 0.01	0.0001
Liver	16.5 ± 7.4	0.0001
Controls	93.3 ± 8.8

**Table 3:** Univariate analysis reporting independent t-test, Impaired (AChE) in cancer makes it a key diagnostic indicator: A hospital-based multicenter case–control study; 2020 (n = 150).

Groups	Mean AChE% activity	*p*-value
AChE in Cancer group	53.4 ± 20.3	0.0001
AChE in patients with no cancer	93.3 ± 8.8

**Table 4:** Multivariate analysis reporting adjusted odds ratio, Impaired (AChE) in cancer makes it a key diagnostic indicator: A hospital-based multicenter case–control study; 2020 (n = 150).

Characteristics	OR	95% CI	*p*-value
Gender			
Male vs. female	1.307	(0.688 – 2.484)	0.4139
Age	1.006	(0.978 – 1.035)	0.6658

## Discussion

The primary objective of this study is to assess the level of (AChE) in cancer patients and evaluate its potential application as a diagnostic biomarker and therapeutic target. Apart from its well-established physiological role in the nervous system, the widely distributed expression of acetylcholine receptors AChRs in different human organs suggests its role in other biological processes. Accumulating evidence has revealed that cancer cell processes such as proliferation, apoptosis, angiogenesis, and epithelial-mesenchymal transition are mediated by the overexpression of AChRs in different types of tumors. ACh enhances breast cancer cell proliferation and estrogen receptor alpha (ERα) activity, this may be due to its contribution in a ligand-independent pathway, which may suggest its proliferative role in the development of breast cancer^30^. In breast cancer, α7-nAChR and α9-nAChR have been reported to be oncogenic^31^. Our study revealed significantly impaired (AChE) in patients with cancer, a finding similar to that reported in a study conducted by (Pedro Martinez-Moreno Susana et al.) who examined the association between impaired (AChE) and lung cancer. They observed a correlation and involvement of (AChE) in cancer, their study also revealed increased availability of acetylcholine in the neoplastic lung resulting from the fall of cholinesterase activity enhancing cholinergic signaling and contributing to tumor progression^32^. Case–control studies were conducted previously to relate (AChE) expression levels and activities in patients having several forms of lung cancers and they showed a significant decrease in AChE activity in such patients^32,33^. Another study reported that (AChE) presents noncholinergic functions that participate in the control of cell proliferation and apoptosis, which are relevant, particularly in hepatocellular carcinoma (HCC) where the results showed a clear relationship between (AChE) expression and cell cycle progression. Human liver tumor samples exhibited a decrease in (AChE) activity compared to normal tissue. The evidence presented herein provides additional support for the proposed tumor suppressor role of (AChE), which makes it a potential therapeutic target in hepatocellular carcinoma as in some patients with liver cancers, hepatic samples showed a reduction in AChE activity in comparison with normal tissue^34^. The roles and implications of (AChE) in hepatocellular carcinoma HCC remain elusive. It has been demonstrated that (AChE) was significantly downregulated in the cancerous tissues of 69.2% of HCC patients, and low (AChE) expression in HCC was correlated with tumor aggressiveness, an elevated risk of postoperative recurrence, and a low survival rate. Both the recombinant (AChE) protein and enhanced expression of (AChE) significantly inhibited HCC cell growth in vitro and tumorigenicity in vivo. Moreover, (AChE) could inactivate the mitogen-activated protein kinase and phosphatidyl inositol-3-phosphate kinase/protein kinase B pathways in HCC cells, thereby increasing the activation of glycogen synthase kinase 3β, leading to β-catenin degradation and cyclin D1 suppression^35^. Our study revealed liver cancer within the severely impaired subgroup of tumors with a mean (AChE) activity of 16.5 ± 7.4. Few published reports have explored the ability of AChE-enhancing agents/inhibitors to sensitize human cancer cells to the pro-apoptotic effects of chemotherapy^36^. In our study, since the majority 55 (72%) of cases were on chemotherapy, and due to the lack of literature on the effect of chemotherapy on (AChE), the potential impairment of (AChE) by chemotherapy is a major concern. Interestingly the impairment of the enzyme in this study ranges from severe in liver cancer to mild in prostate cancer and none of them receive chemotherapy. The results show the total 8 patients with prostate cancer with mean enzyme 62.5 ± 16.4, and the all the 3 patients with liver cancer 16.5 ± 7.4. Low (AChE) activity has a direct effect on the tumor, and a decreased level of AChE activity in turn enhances the local ACh concentrations, leading to more tumoral growth, aggressiveness, and possible metastasis to other tissues^37^. A recent study published September 2023 reported low (AChE) activity increases the local ACh concentrations, causing tumoral growth, aggressiveness, and metastasis observed in different cancer types and tumoral stages, and as tumors develop (AChE) content decreases more due to the overactivation of the PI3k/Akt survival pathway and overexpression of DNMT1^38^. The impaired activity of (AChE) among cancer patients reported in this study and its physiological consequence of ACh accumulation, viewed with the decreased ACh among patients with Alzheimer’s disease (AD) leads to speculation of an intriguing hypothesis for a possible link between these three factors: 1- Impaired AChE/Accumulating Ach, 2- Cancer, and 3- AD. This view is supported by Musicco Adorni et al.^39^, who found the occurrence of both cancer and AD increases exponentially with age in an inverse relationship; the older a person with cancer the reduced risk of AD, and vice versa. A study by Tohgi et al.^40^ showed a significantly lower ACh concentration in patients with AD compared to others. Moreover, the ACh concentration in the cerebrospinal fluid of AD patients is significantly lower in patients with cancer than in controls^41^. The findings of the Ospina-Romero et al. study and three other articles presented an important contribution to the literature on the inverse association between cancer and AD, with growing evidence of a lower risk of cancer among patients with AD compared to patients without AD. Our speculation for some relation between impaired (AChE), cancer, and AD might shed light on the puzzling question of why individuals who have had cancer or go on to develop it perform better on memory testing than those who remain cancer-free^42–45^. Overall, our results of significant impairment of (AChE) in all patients with cancer are similar to the findings by Martínez, who analyzed the cholinergic components from stored fluids and tissues from 35 patients diagnosed with lung cancer and 37 non-cancerous individuals, showing that the measurements of (AChE) activity among cancer patients were significantly lower in cancerous samples than non-cancerous samples with mean and SD values [1.18 ± 0.18 and 1.34 ± 0.17], respectively^33^.

Our study has the strength of providing impaired (AChE) activity in cancer patients. However, it is also important to acknowledge the fundamental limitations of the study: being cross-sectional observational in nature. Additionally, despite the applied 1:1 matching method in selecting the controls to minimize the difference between the two groups. Nevertheless, selection bias remains a potential problem.

## Conclusion

The study reported severe impairment in the activity of the (AChE) in all patients with cancer compared to patients with no cancer. (AChE) may be considered in the future by oncologists as diagnostic biomarker and therapeutic agent. The study recommends larger sample epidemiological studies and experimental molecular studies applying advanced diagnostic devices, including gas chromatography and liquid chromatography which could ideally help in understanding this health problem.

## Data Availability

The dataset supporting the conclusions of this article is included within the article.

## Abbreviations

AChE: Acetylcholinesterase enzyme
ACh: Acetylcholine
CAT: Choline acetyltransferase
ROS: Reactive oxygen stress
RICK: Radiation and Isotopes Center Khartoum
INMO: Institute of Nuclear Medicine and Molecular Biology and Oncology
HCC: Hepatocellular carcinoma
AD: Alzheimer’s disease
